# Medical treatment for heavy menstrual bleeding in primary care: 10-year data from the ECLIPSE trial

**DOI:** 10.3399/bjgp.2022.0260

**Published:** 2022-11-15

**Authors:** Joe Kai, Brittany Dutton, Yana Vinogradova, Nicholas Hilken, Janesh Gupta, Jane Daniels

**Affiliations:** Centre for Academic Primary Care, University of Nottingham, Nottingham.; Centre for Academic Primary Care, University of Nottingham, Nottingham.; Centre for Academic Primary Care, University of Nottingham, Nottingham.; Nottingham Clinical Trials Unit, University of Nottingham, Nottingham.; Centre for Women’s and Newborn Health, University of Birmingham, Birmingham.; Nottingham Clinical Trials Unit, University of Nottingham, Nottingham.

**Keywords:** cohort studies, endometrial ablation techniques, female, hysterectomy, menorrhagia, primary health care, quality of life

## Abstract

**Background:**

Heavy menstrual bleeding (HMB) is a common problem that can significantly affect women’s lives. There is a lack of evidence on long-term outcomes after seeking treatment.

**Aim:**

To assess continuation rates of medical treatments and rates of surgery in women 10 years after initial management for HMB in primary care.

**Design and setting:**

This was a prospective observational cohort study.

**Method:**

Women with HMB who participated in the ECLIPSE primary care trial (ISRCTN86566246) completed questionnaires 10 years after randomisation to the levonorgestrel-releasing intrauterine system (LNG-IUS) or other usual medical treatments (oral tranexamic acid, mefenamic acid, combined oestrogen–progestogen; or progesterone alone). Outcomes were rates of surgery, medical treatments, and quality of life using the 36-item Short-Form Health Survey (SF-36) and EuroQoL EQ-5D.

**Results:**

The responding cohort of 206 women was demographically and clinically representative of the original trial population. Mean age at baseline was 41.9 years (SD 4.9) and 53.7 years (SD 5.1) at follow-up. Over the 10-year follow-up, 60 of 206 (29.1%) women had surgery (hysterectomy *n* = 34, 16.5%; endometrial ablation *n* = 26, 12.6%). Between 5 and 10 years, 89 women (43.2%) ceased all medical treatments and 88 (42.7%) used LNG-IUS alone or in combination with other treatments. Fifty-six women (27.2%) were using LNG-IUS at 10 years. There were improvements over time in quality-of-life scores, with no evidence of differences in these or other outcomes between the two groups.

**Conclusion:**

Medical treatments for women with HMB can be successfully initiated in primary care, with low rates of surgery and improvement in quality of life observed a decade later.

## INTRODUCTION

Heavy menstrual bleeding (HMB) is a common problem that can significantly affect women’s lives until menopause. Although diagnostic definitions using menstrual blood loss exist, it is the impact on a women’s physical, emotional, social, and economic quality of life that guides treatment.^1^^,^^2^

In 2007, the National Institute for Health and Care Excellence (NICE) published guidelines for HMB, updating them in 2018. These recommend starting medical treatment for HMB without investigation if history and/or examination suggest low risk of uterine pathology; or taking account of history and examination, following ultrasound and/or hysteroscopy to exclude this. The levonorgestrel-releasing intrauterine system (LNG-IUS) is recommended as first-line treatment for women with no uterine pathology, or the use of other medical treatments if LNG-IUS is declined or not suitable (tranexamic acid, non-steroidal anti-inflammatory drugs, combined hormonal contraception, oral progestogens).^1^ NICE emphasises clinical consideration be given to comorbidities, presence of fibroids, adenomyosis or endometrial polyps, contraceptive need, and women’s preferences for first-line treatment. If medical treatments fail to provide effective relief, surgical procedures should be considered.^1^

The NICE recommendations were supported by findings from the original ECLIPSE trial, which randomised 571 women, aged 25 to 50 years, presenting to primary care with HMB to either the LNG- IUS or other usual medical treatment (oral tranexamic acid, mefenamic acid, combined oral contraceptive pill, or progesterone alone, chosen as clinically appropriate by the GP and woman) (ISRCTN86566246).^3^ Women’s eligibility for the original trial, and their clinical assessment consistent with current NICE guidance, are detailed in the Supplementary Information S1. The primary outcome was a patient-reported score of the burden of HMB,^4^ assessed over a 2-year period. This improved significantly from baseline in both groups across all timepoints, although the improvements in women in the LNG- IUS group were significantly greater than those assigned usual medical treatment at 2-year follow- up.^3^ By 5-year follow- up, the benefit of LNG-IUS was reduced.^5^ Consequently, NICE also indicated that the usual medical treatments offered in ECLIPSE be considered for women unable or unwilling to use the LNG-IUS.

To the authors’ knowledge, there is no available research on medical treatment of HMB in the longer term in primary care, beyond the 5-year data from the ECLIPSE trial.^5^ Although women’s need for treatment may be expected to change approaching menopause, further evidence is needed to help inform patient and clinical decision making. The primary objective of this study was to assess continuation rates of medical treatments, and rates of surgical interventions, in women 10 years after initial management for HMB in primary care.

**Table:** How this fits in

Heavy menstrual bleeding (HMB) is a common problem and reason to seek treatment in primary care. It is not known how women then fare in the long term, in order to inform patient and clinical decision making. To the authors’ knowledge, this research is the first to report what proportions of women may be expected to continue to use LNG-IUS (Mirena) or other medical treatments (oral tranexamic acid, mefenamic acid, combined oestrogen–progestogen, or progesterone alone), or progress to surgical intervention, a decade after GP treatment for HMB. It shows that medical treatments for women with HMB can be initiated in primary care with low subsequent rates of surgery and improvement in quality of life 10 years later.

## METHOD

**Figure 1.**
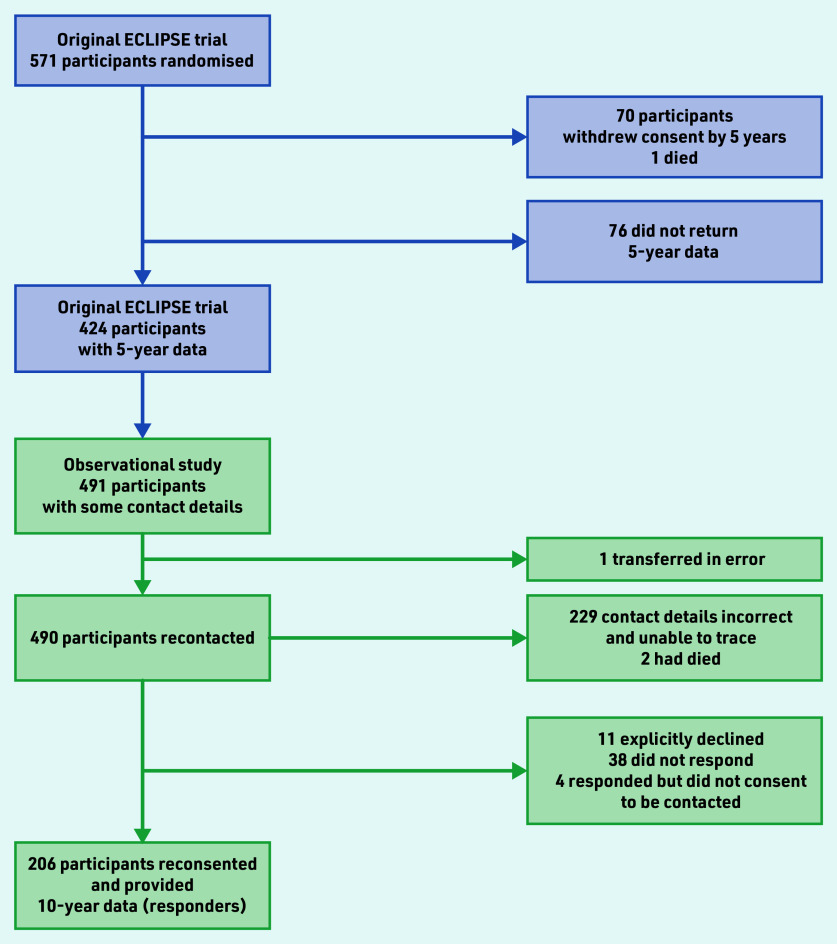
*Progression of participants from the original ECLIPSE trial to the observational study. Attempts to contact women after 23 March 2020 were curtailed because of the COVID-19 pandemic (Supplementary Information S1).*

The ECLIPSE trial ended from a regulatory perspective at 5-year follow-up.^5^ However, data collection continued for this prospective observational study to 10 years. The original trial randomised women between 25 and 50 years of age who presented to their GP with HMB involving at least three consecutive menstrual cycles. The randomisation and interventions used have been previously reported.^3^^,^^5^ Women could subsequently swap or cease their allocated treatment. The aim of the current study was to collect 10-year data from 276 women, equating to 48.3% of the 571 women originally randomised (Figure 1). This target anticipated further loss to follow-up because of the length of time elapsed since previous contact at 2 or 5 years, relocation, non-completion of questionnaire, or death. The process of recontacting and reconsenting participants is described in Supplementary Information S1.

All data were collected directly by questionnaire (paper or via link to online form). The primary outcomes were use of treatments for HMB, and the surgical interventions of hysterectomy and endometrial ablation. Generic quality of life was assessed using the Short-Form Health Survey (SF-36, version 2, with scores ranging from 0 [severely affected] to 100 [not affected]); the EuroQoL EQ-5D descriptive system (with scores ranging from −0.59 [health state worse than death] to 100 [perfect health state]); and the EQ-5D visual analogue scale (with scores ranging from 0 [worst health state imaginable] to 100 [most perfect health state imaginable]). The Sexual Activity Questionnaire (SAQ) measured pleasure (with scores ranging from 0 [lowest level] to 18 [highest level]), discomfort (with scores ranging from 0 [greatest] to 6 [none]), and frequency.^6^ The patient-reported, condition-specific Menorrhagia Multi-Attribute Scale (MMAS)^4^ at 2-year follow-up was the primary outcome for the ECLIPSE trial. As the MMAS only seeks responses in relation to current HMB, completion was optional as it was anticipated to not be relevant to the majority of women at 10-year follow-up. Originally, manual extraction of data on surgical interventions and medical treatments for HMB from patients’ GP records was planned. Twenty-five women from 16 practices reconfirmed consent to this at 10 years. Their self-completed questionnaire data were independently compared by two researchers with their GP-recorded data for completeness and accuracy, which was assessed as very high. Subsequent data extraction from GP records was thus deemed unnecessary unless questionnaire data were missing. However, because of COVID-19 pandemic restrictions no further GP record extraction was performed.

Characteristics of women completing 10-year follow-up were compared with all other women in the original trial cohort (those declining when recontacted or not responding to the recontact invitation). Proportions of women of different ethnicity, HMB presentations, and randomised to different types of treatment were compared using the χ^2^-test. Age in years, body mass index, blood pressure, and questionnaire scores (SF-36, EQ-5D, MMAS, SAQ) in the groups were compared using either Student’s *t*-test for normally distributed variables or the Mann–Whitney test otherwise. Imputation methods for data missing from the SF-36 and SAQ are described in Supplementary Information S1.

The responding cohort of women at 10 years was divided into two subgroups according to their initial randomised treatment allocation. Characteristics and questionnaire scores at baseline and at 10 years of follow-up were compared using the same approach as above. Changes between baseline and 10-year follow-up were assessed using paired *t*-test, whereas changes between groups were examined using an unpaired *t*-test. To compare surgical intervention rates in women allocated to different treatments the log-rank test for equality of survival functions was used and the estimates presented using Kaplan–Meier survival plots

## RESULTS

The progression of women available to be contacted from the original trial to women in the current study (hereafter called responders) are shown in Figure 1. A total of 206 women provided reconsent and returned completed 10-year follow- up data by 31 March 2020 (200 by mail, six online).

The baseline (before randomisation) characteristics of responders and those who were not followed-up are presented in Table 1. Responders were very similar to those women not followed up, with an average age of 41.9 and 41.1 years, respectively, and did not differ clinically in their initial symptoms and presentations of HMB.

**Table 1.** Characteristics and questionnaire scores at baseline (before randomisation in original trial) between responders and women not followed-up at 10 years^a^

**Characteristic**	**All women followed-up at 10 years (*n* = 206)**	**All women not followed-up (*n* = 365)**
**Age at start, years**		
Mean (SD)	41.9 (4.9)	41.1 (5.4)
Age ≥35 years, *n* (%)	188 (91.2)	324 (88.7)

**Ethnicity**, ***n* (%)**		
White	178 (86.4)	293 (80.2)
Asian	11 (5.3)	40 (10.9)
Black	9 (4.4)	21 (5.7)
Other	8 (3.8)	11 (3.0)

**BMI, kg/m^2^**		
Mean (SD)	29.4 (6.4)	29.1 (6.4)
BMI ≥25, *n* (%)	146 (70.8)	255 (69.9)

**Blood pressure**		
Systolic blood pressure, mean (SD)	129.7 (17.0)	128.5 (16.3)
Diastolic blood pressure, mean (SD)	78.8 (10.2)	78.7 (10.5)

**Presentation to primary care for HMB**, ***n* (%)**		
Initial	157 (76.2)	279 (76.4)
Subsequent	49 (23.8)	86 (23.6)
Duration of HMB >1 year	164 (79.6)	296 (81.1)
Menstrual pain	151 (73.3)	273 (74.8)
Contraception requirement	35 (16.9)	75 (20.5)
Copper or non-hormonal coil	7 (3.4)	12 (3.3)

**Treatment at randomisation**, ***n* (%)**		
LNG-IUS	110 (53.4)	175 (47.9)
Usual medical treatments	96 (46.6)	190 (52.1)

**Questionnaire scores, mean (SD) *n***		
SF-36		
Physical functioning	82.5 (19.4) 205	76.2 (24.6) 339
Physical role	71.7 (24.3) 205	69.6 (26.2) 340
Emotional role	72.0 (24.9) 204	70.2 (26.6) 339
Social functioning	65.7 (23.7) 205	61.9 (26.0) 342
Mental health	60.7 (19.6) 205	59.1 (19.5) 340
Energy and vitality	40.8 (21.9) 205	40.7 (20.9) 340
Pain	48.5 (22.6) 205	45.6 (22.3) 342
Perception of general health	62.2 (21.8) 205	60.2 (21.7) 342
EuroQoL EQ-5D		
Descriptive system	0.769 (0.228) 206	0.714 (0.276) 340
EQ-5D visual analogue scale	71.6 (18.9) 185	69.0 (19.7) 311
Sexual Activity Questionnaire		
Pleasure	10.5 (5.0) 166	11.1 (4.9) 248
Discomfort	4.8 (1.4) 166	4.5 (1.7) 248
Menorrhagia Multi-Attribute Score	42.8 (19.4) 206	39.7 (21.8) 206

a *If information was partially missing but over half of questions in a domain were answered; the average score of the responses was used, otherwise they were classed as missing. The mean scores and number of contributing participants are slightly different from the original ECLIPSE trial because of this method. BMI = body mass index. HMB = heavy menstrual bleeding. LNG-IUS = levonorgestrel-releasing intrauterine system. SD = standard deviation. SF-36 = 36-item Short-Form Health Survey.*

Allocation to different treatments was balanced across both groups of women: 110 of 206 (53.4%) responders and 175 of 365 (47.9%) of women not followed up were allocated to LNG-IUS. Responders and those not followed up also had similar baseline scores for SF-36, EQ-5D, and SAQ, with no domains showing a statistically significant difference (Table 1). Average scores at baseline for MMAS were slightly higher for women responding at 10 years (42.8 versus 39.7), and the difference was not statistically significant.

The 206 responders had a mean age at response to the 10-year follow-up of 53.7 years (SD 5.1 years) and 178 (86.4%) were of White ethnicity. Among these, 110 were originally allocated to the LNG-IUS and 96 were allocated to other usual medical treatment. At the time of completing the 10-year follow-up questionnaire, 106 (51.5%) women had reached menopause (defined for the responders as having experienced no menstrual bleeding for at least 1 year) and 34 (16.5%) had had a hysterectomy (Table 2). Twelve women (5.8%) were still experiencing HMB and did not consider themselves menopausal.

**Table 2.** Menopausal status and reported treatments for HMB among all responders, and by original ECLIPSE trial allocations over 10-year follow-up period

	**All responders, *n* (%) (*n* = 206)**	**Allocated to LNG-IUS, *n* (%) (*n* = 110)**	**Allocated to usual medical treatment, *n* (%) (*n* = 96)**
**Menopausal status**			
Premenopausal	32 (15.5)	16 (15.5)	16 (16.6)
Postmenopausal	106 (51.5)	54 (49.1)	52 (54.1)
Undergone hysterectomy	34 (16.5)	18 (16.4)	16 (16.6)
Perimenopausal or uncertain	32 (15.5)	21 (19.1)	11 (11.4)
Missing	2 (0.9)	1 (0.9)	1 (1.0)
Using menopausal hormone therapy	28 (13.6)	16 (14.5)	12 (12.5)
Still experiencing heavy menstrual bleeding	12 (5.8)	6 (5.5)	6 (6.3)
Using LNG-IUS at response to 10-year follow-up	56 (27.2)	38 (34.5)	18 (18.8)

**Classes of treatments used between 5 and 10 years**			
LNG-IUS	67 (32.5)	47 (42.7)	20 (20.8)
Usual medical treatment	29 (14.1)	10 (9.1)	19 (19.8)
LNG-IUS and usual medical treatment	21 (10.2)	11 (10.0)	10 (10.4)
None	89 (43.2)	42 (38.2)	47 (48.9)

**Standard medical treatments used between 5 and 10 years**			
Tranexamic acid	24 (11.6)	7 (6.4)	17 (17.7)
Mefenamic acid	6 (2.9)	3 (2.7)	3 (3.1)
Norethisterone	13 (6.3)	4 (3.6)	9 (9.3)
Desogestrel	3 (1.4)	0	3 (3.1)
Oral contraceptives	8 (3.9)	3 (2.7)	5 (5.2)
Medroxyprogesterone acetate injection	1 (<1)	1 (<1)	0
Naproxen	1 (<1)	0	1 (<1)

**Surgical intervention for HMB**			
Hysterectomy	34 (16.5)	18 (16.4)	16 (16.6)
Endometrial ablation	26 (12.6)	10 (9.1)	16 (16.6)

*HMB = heavy menstrual bleeding. LNG-IUS = levonorgestrel-releasing intrauterine system.*

Between 5 and 10 years of follow-up, a substantial proportion of women (89, 43.2%) reported not taking treatments for HMB. However, 88 (42.7%) of women used LNG-IUS (67 women used only LNG-IUS, and 21 used LNG-IUS in combination with usual medical treatment). The proportions using LNG-IUS, alone or in combination, were higher for women initially allocated to LNG-IUS than to usual medical treatment (58/110 women [52.7%] and 30/96 women [31.3%], respectively).

Overall, 56 (27.2%) women reported they were using LNG-IUS at the time of response to the 10-year follow-up (including 34.5% [38/110] of women originally allocated to LNG-IUS and 18.8% [18/96] of women originally allocated to medical treatments). Table 2 shows the reported treatments by original randomised allocation. There were no statistically significant differences in treatments between the two randomised groups for any menopausal or treatment category.

Table 3 reports the distributions of SF-36, EQ-5D, and SAQ scores, for all responders and by the original randomised allocation, 10 years after randomisation. There were no statistically significant differences between the randomised groups in any domain of the three questionnaires. Only 13 responders, 12 of whom described their bleeding as heavy, completed the MMAS questionnaire, so distributions were not calculated, nor groups compared. The SAQ was completed by 116 of the 206 responding women, indicating at least 56.3% of women were sexually active.

**Table 3.** Questionnaire scores at 10 years among all responders, and by original ECLIPSE trial allocations

	**All responders, mean (SD) *n***	**Allocated to LNG-IUS, mean (SD) *n***	**Allocated to usual medical treatment, mean (SD) *n***
**SF-36**			
Physical functioning	80.2 (26.2) 205	81.4 (24.9) 110	78.8 (27.7) 95
Physical role	78.4 (28.6) 204	80.1 (26.2) 109	76.4 (31.1) 95
Emotional role	79.4 (27.5) 204)	79.3 (26.4) 109	79.5 (28.9) 95
Social functioning	74.7 (25.8) 206	75.5 (25.2) 110	73.8 (26.6) 96
Mental health	68.6 (21.5) 205	68.1 (21.1) 110	69.2 (22.0) 95
Energy and vitality	48.9 (10.2) 205	48.3 (8.8) 110	49.5 (11.6) 95
Pain	63.4 (24.8) 206	64.3 (23.9) 110	62.4 (25.9) 96
Perception of general health	55.4 (9.6) 206	55.9 (10.3) 110	54.9 (8.7) 95

**EuroQoL EQ-5D**			
Descriptive system	0.748 (0.266) 204	0.757 (0.249) 110	0.736 (0.286) 94
Visual analogue scale	73.4 (20.7) 176	74.9 (19.8) 93	71.8 (21.6) 83

**Sexual Activity Questionnaire**			
Pleasure	11.2 (4.6) 116	11.5 (4.6) 62	10.9 (4.6) 54
Discomfort	2.01 (1.99) 116	2.19 (2.09) 62	1.80 (1.87) 54

*LNG-IUS = levonorgestrel-releasing intrauterine system. SD = standard deviation. SF-36 = 36-item Short-Form Health Survey.*

Table 4 presents scores for these three questionnaires by randomised group at baseline and at 10-year follow-up, including only those women who completed questionnaires at both timepoints.

**Table 4.** Questionnaire scores at baseline and 10 years of follow-up^a^

	**Baseline scores for responders, mean (95% CI)**		**10-year follow-up, mean (95% CI)**		**Difference between groups over 10 years, mean (95% CI) *P*-value**	**Change within group, mean (95% CI) *P*-value**	
		
	**LNG-IUS**	**Usual medical treatment**	**LNG-IUS**	**Usual medical treatment**		**LNG-IUS**	**Usual medical treatment**
**SF-36**							
Physical functioning	84.0 (81.5 to 86.5)	80.7 (77.8 to 83.6)	81.2 (78.2 to 84.2)	78.8 (75.5 to 82.1)	−0.9 (−4.4 to 2.6) 0.786	−2.8 (−5.7 to 0.2) 0.220	−1.9 (−4.9 to 1.1) 0.409
Physical role	74.0 (71.0 to 76.9)	69.1 (65.9 to 72.2)	79.9 (76.8 to 83.0)	76.4 (72.9 to 79.9)	−1.3 (−5.4 to 2.8) 0.760	6.0 (2.7 to 9.3) 0.038	7.3 (3.7 to 10.9) 0.034
Emotional role	72.4 (69.4 to 75.5)	71.2 (68.1 to 74.4)	79.8 (76.8 to 82.9)	79.5 (76.1 to 82.8)	−0.8 (−4.9 to 3.2) 0.844	7.4 (4.2 to 10.6) 0.007	8.2 (4.6 to 11.9) 0.018
Social functioning	67.2 (64.4 to 70.0)	64.1 (60.9 to 67.3)	75.2 (72.2 to 78.3)	73.8 (70.6 to 77.1)	−1.7 (−5.6 to 2.2) 0.661	8.0 (5.0 to 11.0)<0.001	9.8 (6.2 to 13.3) 0.004
Mental health	61.7 (59.0 to 64.4)	60.0 (57.3 to 62.8)	68.1 (65.3 to 70.9)	69.2 (66.2 to 72.1)	−2.8 (−6.1 to 0.5) 0.331	6.3 (3.7 to 9.0) <0.001	9.1 (6.2 to 12.1)<0.001
Energy and vitality	41.6 (38.8 to 44.4)	40.0 (37.0 to 43.0)	48.3 (46.5 to 50.1)	49.5 (47.4 to 51.7)	−2.8 (−6.4 to 0.7) 0.392	6.7 (3.8 to 9.6) 0.003	9.5 (6.5 to 12.6)<0.001
Pain	49.0 (46.1 to 51.9)	47.9 (44.9 to 50.9)	64.1 (61.2 to 67.1)	62.4 (59.2 to 65.6)	0.7 (−3.2 to 4.5) 0.866	15.1 (12.0 to 18.3) <0.001	14.5 (11.1 to 17.8) <0.001
Perception of general health	63.5 (60.7 to 66.3)	60.7 (57.7 to 63.6)	56.0 (54.0 to 57.9)	54.9 (53.0 to 56.7)	−1.8 (−5.2 to 1.7) 0.564	−7.5 (−10.4 to −4.7) <0.001	−5.8 (−8.7 to −2.8) 0.011

**EuroQoL EQ-5D**							
Descriptive system	0.78 (0.50 to 1.07)	0.75 (0.44 to 1.06)	0.76 (0.46 to 1.06)	0.74 (0.40 to 1.07)	−0.01 (−0.39 to 0.37) 0.782	−0.03 (−0.33 to 0.28) 0.270	−0.02 (−0.36 to 0.33) 0.607
Visual analogue scale	73.5 (70.7 to 76.3)	70.3 (67.3 to 73.3)	76.2 (73.5 to 78.9)	72.3 (69.3 to 75.4)	0.7 (−2.9 to 4.3) 0.832	2.8 (−0.2 to 5.7) 0.214	2.0 (−1.1 to 5.2) 0.442

**Sexual Activity Questionnaire**							
Pleasure	11.8 (10.3 to 13.3)	10.4 (8.6 to 12.1)	11.3 (9.7 to 12.8)	10.9 (9.3 to 12.5)	−1.1 (−3.2 to 1.0) 0.323	−0.5 (−2.3 to 1.2) 0.487	0.6 (−1.2 to 2.3) 0.482
Discomfort	4.6 (3.8 to 5.5)	5.0 (4.1 to 5.8)	2.3 (1.3 to 3.4)	1.7 (0.8 to 2.7)	0.9 (−0.5 to 2.3) 0.075	−2.3 (−3.5 to −1.1) <0.001	−3.2 (−4.4 to −2.1) <0.001

a *Scores are only calculated for women who provided both baseline and 10-year data, so the baseline scores are slightly different from those reported for the original ECLIPSE trial and in Table 3 where all scores are reported. CI = confidence interval. LNG-IUS = levonorgestrel-releasing intrauterine system. SF-36 = 36-item Short-Form Health Survey.*

There were improvements over time in SF-36 scores in all domains, except general health perception and physical functioning, and in EQ-5D scores. These improvements occurred in both groups, with small and statistically insignificant differences between groups.

Of the 206 women, 40 were not in an intimate relationship and 116 reported via the SAQ that they were sexually active. There was a clear deterioration within the discomfort domain of the SAQ, although with no evidence of a difference between the allocation groups, but no changes were seen within the pleasure domain.

### Surgical interventions

**Figure 2.**
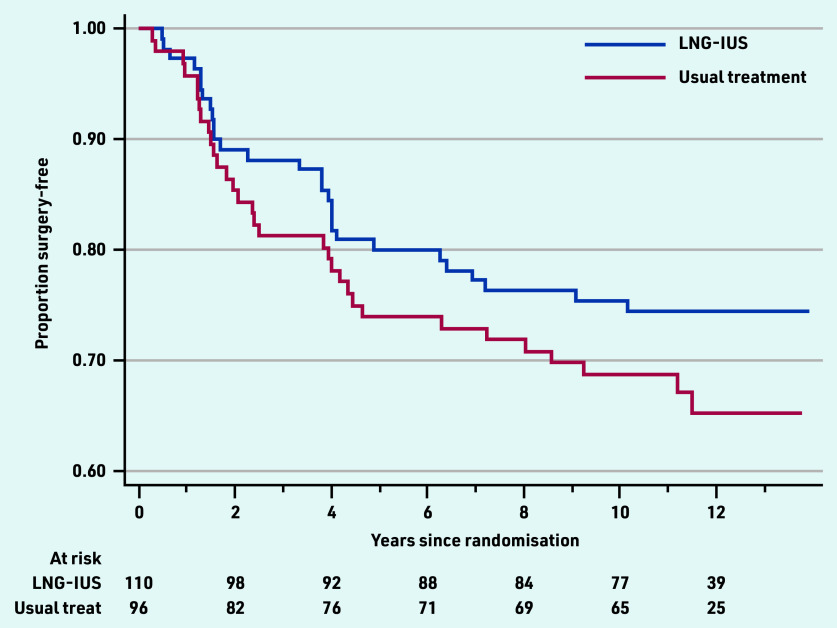
*Surgery-free time for all responders by original ECLIPSE trial allocations. LNG-IUS = levonorgestrel-releasing intrauterine system.*

Over the 10-year follow-up period, there were 60 of 206 (29.1%) women who had had surgical intervention, including hysterectomy (*n* = 34, 16.5%) or endometrial ablation (*n* = 26, 12.6%). No woman had both procedures and no one who had a surgical procedure reported HMB at 10 years. The cumulative rate of surgery was slightly lower in women initially allocated to LNG-IUS (28/110 women, 25.5%) compared with those allocated to standard medical treatment (32/96, 33.3%), (data not shown) in the ECLIPSE trial. Considering the opposite outcome, the surgery-free rate, including all data collected over a median of 11.2 years, the cumulative surgery-free rate was 74% for LNG-IUS and 65% for usual medical treatment, shown in Figure 2, and the difference was not statistically significant (hazard ratio 0.73, 95% confidence = 0.44 to 1.21, *P* = 0.22).

## DISCUSSION

### Summary

This study shows medical treatments for women with HMB can be initiated in primary care with improvement in quality of life and high likelihood of avoiding surgery 10 years later. Among women, typically presenting with HMB in their early forties, this study found that half reach the menopause in the ensuing decade and over 40% may be expected to cease medical treatments over this time. However, a similar proportion (42.7%) continue to use LNG-IUS alone or in combination with other oral treatments, and almost 30% were using LNG-IUS after 10 years.

Relatively low rates of surgical intervention were sustained at 29% after 10 years, modestly increased from those at 5 (around 20%) and 2 (around 10%) years after commencing treatment in primary care.^3^^,^^5^ Women initially treated with LNG- IUS were slightly less likely to need surgical intervention than those commenced on standard medical treatments; however, this was not statistically or clinically significant. There were improvements over time in generic quality-of-life scores in both women who were initially allocated LNG-IUS or to other usual medical treatment, but with no evidence of any significant differences between the two original groups.

### Strengths and limitations

This research has ascertained outcomes in women a decade after initial treatment for HMB in primary care, following participation in the largest trial of medical treatments for HMB.^3^^,^^5^ Responses were achieved from 206 women, 206/571, 36.1% of the original trial population and 206/490, 42.0% of those potentially available for recontact after 10 years. Although this was lower than anticipated because of difficulties during the height of the COVID-19 pandemic, such long-term data for women with HMB, to the authors’ knowledge, have not been available before, nor at this scale. Responding women were very similar, both demographically and clinically at presentation, to non-responders, lending confidence in the generalisability of the trajectories reported.

The original trial and current study follow-up population reflect the ethnic diversity of England and Wales when women were recruited (87% White, 13% Black/Asian/Other in 2011 UK census). However, it is recognised that further research with women from Black and ethnic minority communities is needed as HMB experiences may differ, especially given the higher prevalence of fibroids in Black women.^7^

Given the proportion of participants who had changed or ceased their original allocated treatments by 5 years, it was anticipated that intention-to-treat comparisons at 10 years would have limited ability to demonstrate a difference for the participant-reported quality-of-life instruments. A large proportion of women had, as expected, stopped having periods, either because of the menopause, or surgical treatment, meaning few women were able to report on the original primary outcome measure, the MMAS. Nevertheless, it has been possible to illustrate for the first time the proportion of women progressing to surgical intervention by initial medical treatment.

The original intention had been to collect data from GP records, but cross-checking against women’s self-reported data suggested this did not add value. As GP practices then became inaccessible to researchers during the COVID-19 pandemic, the potential for missing data exists but is probably limited. Women’s own knowledge and reporting of whether they had an LNG-IUS in situ or not, their use of other oral medical treatments, their perception of being perimenopausal or of having surgery, is likely to be accurate and using this in the current study was the most realistically achievable option. Participating women’s qualitative experiences of HMB and influences on their treatment over time will be reported separately in a future article.

### Comparison with existing literature

To the authors’ knowledge, this study is the first to report outcomes a decade after commencing medical treatment for HMB in primary care. Evidence from a secondary care trial comparing LNG-IUS with hysterectomy followed 119 women allocated LNG-IUS, reporting 55 (46%) had had a hysterectomy, 44 (37%) were still using LNG-IUS, one had had endometrial ablation, and 18 were not using LNG-IUS after 10 years.^8^ The higher rate of hysterectomies can be attributed to women’s recruitment from a hysterectomy waiting list. In the current study, as the starting point was initial medical treatment, there were too few women who had had endometrial ablation to determine the rate of subsequent procedures: previous evidence suggests around 20% of women need further surgery.^9^

There are no recent UK data to suggest a change in patterns of treatments for HMB. Drug utilisation data in Denmark between 1996 and 2017 showed a large increase in use of LNG-IUS (from 2.3 to 32 users per 1000 person-years) and a decline in use of oral tranexamic acid (from 11.3 to 6.3 per 1000 person-years) for women aged 20–54 years. Use of combined hormonal contraceptives remained stable, and use of cyclical oral progestogens decreased.^10^ Dutch data between 2004 and 2013 show progestogen prescriptions also declining over time, although LNG-IUS was used in less than 2.5% of patients.^11^

### Implications for practice

The original ECLIPSE trial recruited women from the general population who had HMB that was affecting their lives, who chose to present to their GP with this problem, and who were clinically assessed as appropriate for, and who wanted to have, medical treatment. This assessment and the range of medical treatments used (LNG-IUS or other standard medical treatments) reflected real-life practice and remains the range of choices available to women of any age and their GP in the community setting, according to women’s individual needs and preferences. This is consistent with current updated NICE guidance for initial management of HMB.^1^

The sustained low rates of progression to surgical intervention observed, and general improvement in quality of life, 10 years from women’s initial presentation, underline the importance and value of initiating medical management of women’s HMB in primary care, where most women seek help from health services. Avoiding referrals to secondary care is likely to reduce operative intervention rates. The findings provide helpful information for women and GPs on what to expect in the longer term from starting treatments for HMB and to inform individual decision making. This includes women’s chances of surgery, of continuing or ceasing medical treatments, and an accurate estimate of 10-year retention of LNG-IUS.

Wider public awareness is also needed to encourage women to seek help for HMB if it is affecting their lives, as they are likely to benefit from treatments commenced in the community setting. Ongoing care should ensure clinical willingness to continue review of women’s response, their working diagnosis, need for further investigation or different treatment, or surgical options over time. This should include counselling in those women considering removal or renewal of LNG-IUS at 5 years so that they may continue to benefit and avoid surgery.

In conclusion, the study provides a helpful new indication of expected proportions of women continuing to use or not use treatments for HMB, or progressing to surgical intervention, and of the significant proportion of women using LNG-IUS after a decade. Medical treatments for women with HMB can be initiated in primary care with low rates of surgical intervention and improvement in quality of life observed 10 years later. The study supports current NICE recommendations^1^ on medical management of HMB, and confirms many women with HMB do not require surgery as there are less invasive and acceptable alternatives.

## Data

Data requests for 10-year data should be submitted to the corresponding author for consideration. Access to available anonymised data may be granted following review. Data requests for the original ECLIPSE trial dataset should be submitted to bctudatashare@bham.ac.uk.
